# Preventive Chemotherapy Versus Innovative and Intensified Disease Management in Neglected Tropical Diseases: A Distinction Whose Shelf Life Has Expired

**DOI:** 10.1371/journal.pntd.0004521

**Published:** 2016-04-14

**Authors:** Mark Rosenberg, Jürg Utzinger, David G. Addiss

**Affiliations:** 1 The Task Force for Global Health, Decatur, Georgia, United States of America; 2 Swiss Tropical and Public Health Institute, Basel, Switzerland; 3 University of Basel, Basel, Switzerland; Institute of Tropical Medicine (NEKKEN), JAPAN

Dr. Julio Frenk, the former dean of the Harvard School of Public Health, has said (in a personal communication) that “The greatest threat to global health is dichotomous thinking, thinking that things are either black or white, all good or all bad.” This sort of thinking is simplistic and often prevents us from seeing how to best solve our most serious problems, pitting advocates against each other and blinding different stakeholders to innovations and solutions that involve collaboration, dialogue, and compromise.

The term “neglected tropical diseases” (NTDs) came into use some ten years ago to refer to a group of diseases that occur most commonly in the tropics and subtropics [1,2]. Although NTDs cause considerable morbidity and human suffering, progress in controlling them remained limited, in large part because they affect marginalized and impoverished populations. The NTD label provided a greater profile, facilitated advocacy, and, especially in the last couple of years, caught the attention of policy makers, philanthropists, and donors [3].

For a number of years, we have used a distinction between those NTDs whose treatment and prevention involve mass drug administration (MDA) and those which involve individual case finding and case management. We have labeled the former the “preventive chemotherapy and transmission control” (PCT) NTDs and the latter the “innovative and intensified disease management” (IDM) NTDs. The Department of Control of Neglected Tropical Diseases of the World Health Organization (WHO), for example, uses this distinction and accordingly has classified some diseases as “IDM” and others as “PCT” (see: http://www.who.int/neglected_diseases). Most people working in the NTD field have accepted and adhere to this distinction.

PCT focuses on those NTDs for which a global strategy exists and for which tools are readily available for large-scale deployment [4]. The most prominent examples of NTDs that have been allocated to the PCT group are lymphatic filariasis, onchocerciasis, schistosomiasis, and soil-transmitted helminthiasis; the main tool for their control is the periodic administration of efficacious, safe, and inexpensive (usually donated) drugs to entire at-risk populations without prior individual diagnosis [1,2,5,6]. IDM, on the other hand, focuses on those NTDs that currently lack appropriate tools for large-scale use. Hence, in general, the IDM NTDs are currently more difficult and costly to manage than some of the PCT diseases, as there are inherent challenges for their diagnosis, treatment, and follow-up. Buruli ulcer [7], Chagas disease [8], human African trypanosomiasis [9], and leishmaniasis [10] are examples of IDM NTDs. The separation of NTDs into mutually exclusive groups has been further cemented by referral to “tool-ready” and “tool-deficient” diseases—a terminology used by WHO [4] and the Centers for Disease Control and Prevention (CDC; see: http://www.cdc.gov/globalhealth/ntd/diseases/), among other organizations.

In our view—and that of others [11]—the distinction between PCT and IDM and between “tool-ready” and “tool-deficient” NTDs needs careful rethinking. Indeed, we feel that the shelf life of this separation has expired. For example, look at the case of yaws. Yaws is an NTD caused by a spirochete related to the organism that causes syphilis [12]. The traditional approach to address yaws was to identify persons with the characteristic skin ulcers and treat them with injectable penicillin, together with skin care and careful follow-up. But recently, it has been shown that mass administration of oral azithromycin can cure almost all cases of yaws and effectively stop transmission [13]. This means that a disease formerly classified as an IDM NTD might be reclassified as a PCT NTD. It must be noted, however, that not all yaws-like lesions are, in fact, yaws. Careful diagnosis is needed to distinguish them and provide appropriate case management. Further, yaws leg ulcers will often persist after azithromycin treatment, and there will be a need to find such patients for follow-up. Taken together, yaws eradication will require both MDA and case identification as well as rigorous surveillance and adequate responses tailored to specific social-ecological contexts.

Other so-called PCT NTDs are not so clear-cut as to fit neatly into either one or the other category. Lymphatic filariasis, for example, can be prevented with MDA, but millions of people who already have filariasis-associated lymphedema require careful case management, including scrupulous hygiene and skin care, which may need to be lifelong [14,15]. Such case management reduces the frequency of secondary bacterial infections and arrests, reverses, or slows progression of disease [14,15]. Additionally, surgery is required to address the massive burden of filariasis-associated hydrocele [16]. Putting lymphatic filariasis into the PCT NTD box has diminished the importance of case management and resulted in this crucial component of lymphatic filariasis elimination being neglected and downplayed. Lymphatic filariasis may also be prevented by the use of mosquito netting, and this strategy, too, gets obscured when lymphatic filariasis is termed a PCT disease [17].

Trachoma is another disease typically categorized as a PCT NTD [18]. The WHO “SAFE” strategy for eliminating blinding trachoma has four parts: surgery for trichiasis (the S component of the strategy), antibiotic mass distribution (A component), facial cleanliness (F component), and environmental improvement (E component). When trachoma is labeled a PCT NTD, the other critical parts of the strategy become easily forgotten and ignored. In places where blinding trachoma has disappeared in concert with economic development, it is conceivable that these non-MDA parts of the strategy have played a pivotal role.

Schistosomiasis and soil-transmitted helminthiasis are NTDs for which water, sanitation, and hygiene (WASH) are extremely important components of the prevention and control strategy [19,20]. Indeed, in the absence of WASH, there is rapid “reworming” after people have been treated with deworming drugs [21]. Improved access to and use of sanitation is associated with lower odds of infection with soil-transmitted helminths [20,22]. The role of WASH is particularly relevant when the goal is elimination of transmission [23].

Compared to the PCT NTDs, advocates for controlling IDM NTDs have had a harder time attracting resources and developing and delivering implementation strategies. One might speculate that adherence to a PCT versus IDM distinction might have contributed to lack of awareness of IDM diseases and the scarcity of resources for innovation, research, and development. Moreover, with success in interrupting or significantly reducing transmission, MDA for PCT NTDs such as lymphatic filariasis is being scaled back. The post-transmission era—for lymphatic filariasis and for other NTDs approaching elimination—will require enhanced surveillance in order to rapidly detect pockets of recrudescent or ongoing transmission and initiate targeted responses. Taken together, the initial focus of this PCT NTD is shifting to an IDM NTD, which again renders the distinction into the two categories problematic. What’s more, now that MDA with ivermectin is being tried as a strategy for eliminating malaria, the question arises whether malaria should be labeled a PCT disease [24]. Would that be helpful? What might the impact of doing so be on the use of long-lasting insecticidal nets, indoor residual spraying, and mosquito larval control? And into which category will we place those NTDs for which vaccines will become available?

Finally, there is increasing recognition of the need to strengthen health systems and the importance of intersectoral collaboration in tackling the NTDs [25,26]. The sectors that need to play a role include urban development, water and sanitation, clinical health care and public health, transportation, and agriculture, among others. It will be much harder to achieve real collaboration among the different sectors if our language implies that the non-health sectors are merely contributing ancillary, nonessential activities [27]. Moreover, people must have access to curative drugs in health facilities (e.g., albendazole or mebendazole for soil-transmitted helminthiasis and praziquantel for schistosomiasis) so that symptomatic patients can be treated at any time and not just during MDA campaigns, perhaps once a year. Thus, PCT NTDs become IDM NTDs that can be managed on a daily basis through strengthened health systems where essential medicines are available.

We believe it is time to seriously rethink the practice of dividing NTDs into the allegedly dichotomous categories of PCT versus IDM diseases because the unintended consequences—the “collateral damage”—of using them is starting to outweigh the benefits. We are not saying that the label of PCT NTD has no value. Instead, we are saying that these labels should be used thoughtfully and carefully while we search for better ways to group and label these diseases. We believe that this dichotomy represents the threat that Julio Frenk was talking about. We can do better.
